# Supplementary material for: Insights into the dynamic trajectories of protein filament division revealed by numerical investigation into the mathematical model of pure fragmentation
Source: PLoS Comput Biol. 2021 Sep 3;17(9):e1008964. doi: 10.1371/journal.pcbi.1008964 (PMC8462728; doi:10.1371/journal.pcbi.1008964)
Supplement: S1 Appendix — (PDF) [file pcbi.1008964.s001.pdf]

S1 Appendix. Recovering γ from the data: comparison with [8]

We detail here the method used in [8] to extract γ from the data. We recall that $M_1(t)$ is the number average molecular weight, i.e. the average length of fibrils (the authors of [8] use the classical notation $M_n(t)$). We have, with the notations from

$$M_1(t) = \frac{\displaystyle\int_0^\infty xu(t,x)dx}{\displaystyle\int_0^\infty u(t,x)dx}. \tag{28}$$

Since the integral of $x \to u(t,x)x$ is constant and equal to 1, we have

$$\frac{d}{dt}M_1(t) = -\frac{\displaystyle\frac{d}{dt}\int_0^\infty u(t,x)dx}{\displaystyle\int_0^\infty u(t,x)dx}M_1(t). \tag{29}$$

The fragmentation model (3) gives

$$\frac{d}{dt}\int_0^\infty u(t,x)dx = \int_0^\infty B(x)u(t,x)dx, \tag{30}$$

and the authors of [8] make the approximation (valid in a monodisperse suspension)

$$\int_0^\infty B(x)u(t,x)dx \approx B(M_1(t))\int_0^\infty u(t,x)dx. \tag{31}$$

Hence

$$\frac{d}{dt}M_1(t) \approx -B(M_1(t))M_1(t) = -\alpha\left(M_1(t)\right)^{\gamma+1}. \tag{32}$$

Directly from the formula above the authors get

$$\frac{1}{(M_1(t))^\gamma} = \frac{1}{(M_1(0))^\gamma} + \alpha\gamma t. \tag{33}$$

The authors then notice that a plot of $(M_1(t))^{-\gamma}$ versus reaction time should give a straight line with slope $\alpha\gamma$ and an axis intercept of $(M_1(0))^{-\gamma}$. The authors plot then the curves $t \to (M_1(t))^{-\gamma}$ for several values of γ and check for each value if the curve is linear with respect to time. They determine then the value of γ by a best-fit determination argument.

The main differences with our method are that 1) we do not need a step of best-fit search since we directly read γ as a slope of a curve in log-log scale, and 2) we use some theoretical information on the convergence to an asymptotic profile, whereas their argument is that for large time, the suspension is monodisperse since it concentrates around $x = 0$.
