# Supplementary material for: Insights into the dynamic trajectories of protein filament division revealed by numerical investigation into the mathematical model of pure fragmentation
Source: PLoS Comput Biol. 2021 Sep 3;17(9):e1008964. doi: 10.1371/journal.pcbi.1008964 (PMC8462728; doi:10.1371/journal.pcbi.1008964)
Supplement: S1 Fig — (PDF) [file pcbi.1008964.s002.pdf]

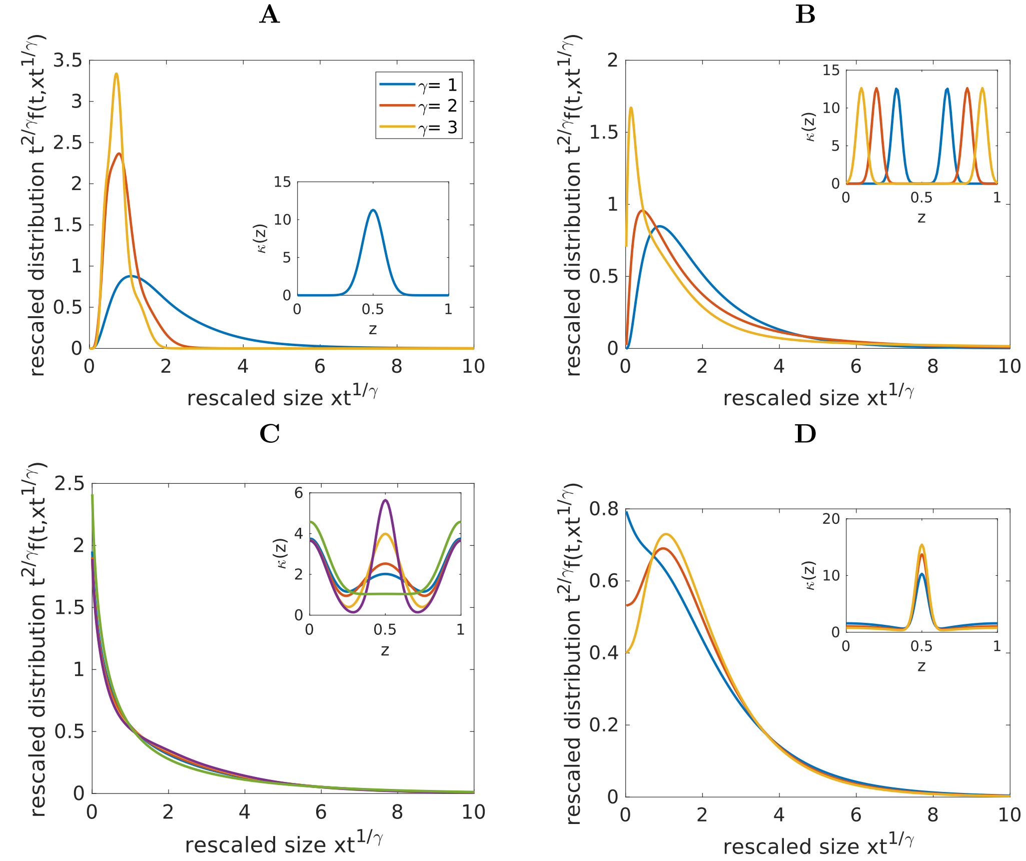

S 1. Influence of the parameters γ and κ on the stationary length distribution profile. A: Stationary profile for various values of γ. For larger values of γ the decrease at infinity is faster. Parameters: $\alpha = 1$, $t_f = 100$. The kernel κ used is plotted in the inset panel. B: Stationary profile for some selected kernels of class A. Parameters: $\gamma = \alpha = 1$, and $t_f = 120$. C and D: Stationary profile for some selected kernels of class B. Even if there is some mass around $z = 1/2$, the asymptotic profile is decreasing for $\kappa(0)$ large enough. Parameters: $\gamma = \alpha = 1$, and $t_f = 40$. For all the figures, it has been checked that stationary state is reached.
