# Supplementary figures and images for: Insights into the dynamic trajectories of protein filament division revealed by numerical investigation into the mathematical model of pure fragmentation
Source: PLoS Comput Biol. 2021 Sep 3;17(9):e1008964. doi: 10.1371/journal.pcbi.1008964 (PMC8462728; doi:10.1371/journal.pcbi.1008964)

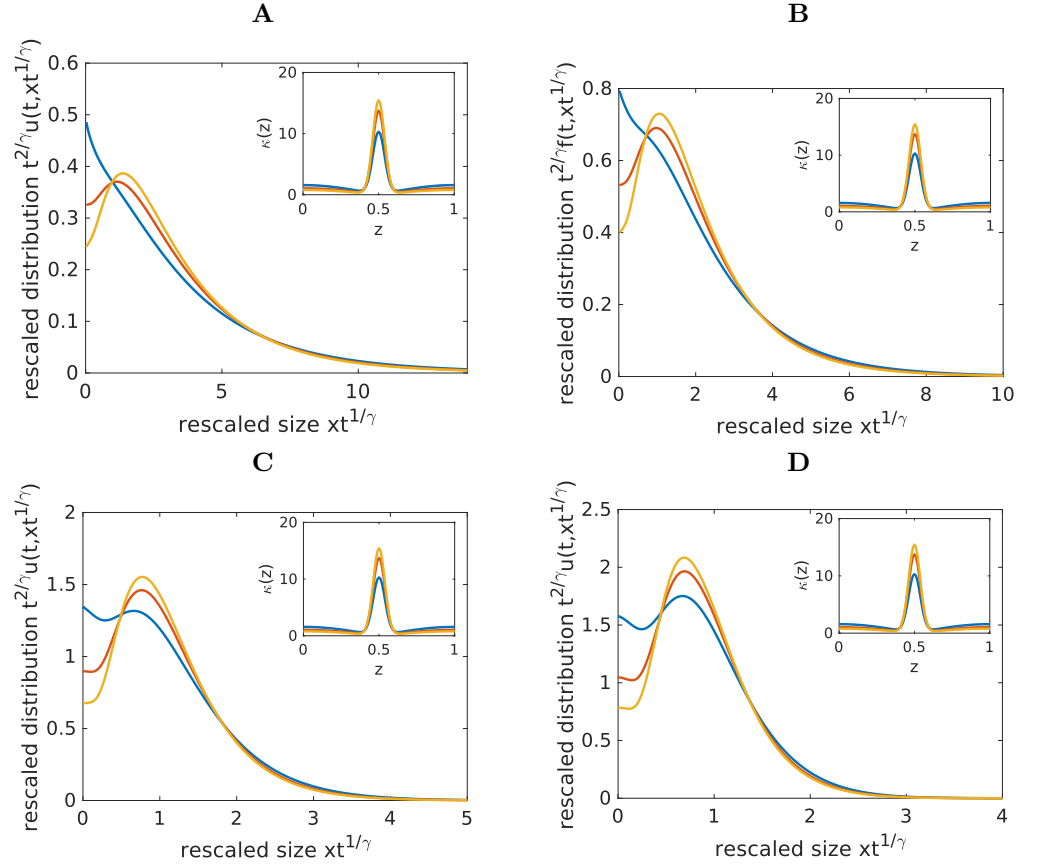

**S 2. Stationary profile for different values of  $\gamma$  and  $\alpha = 1$ .** A:  $\gamma = 0.8$ , B:  $\gamma = 1$ , C:  $\gamma = 1.5$ , D:  $\gamma = 2$ .

Supplement: S2 Fig — (PDF) [file pcbi.1008964.s003.pdf]
