# Supplementary material for: Insights into the dynamic trajectories of protein filament division revealed by numerical investigation into the mathematical model of pure fragmentation
Source: PLoS Comput Biol. 2021 Sep 3;17(9):e1008964. doi: 10.1371/journal.pcbi.1008964 (PMC8462728; doi:10.1371/journal.pcbi.1008964)
Supplement: S3 Fig — (PDF) [file pcbi.1008964.s004.pdf]

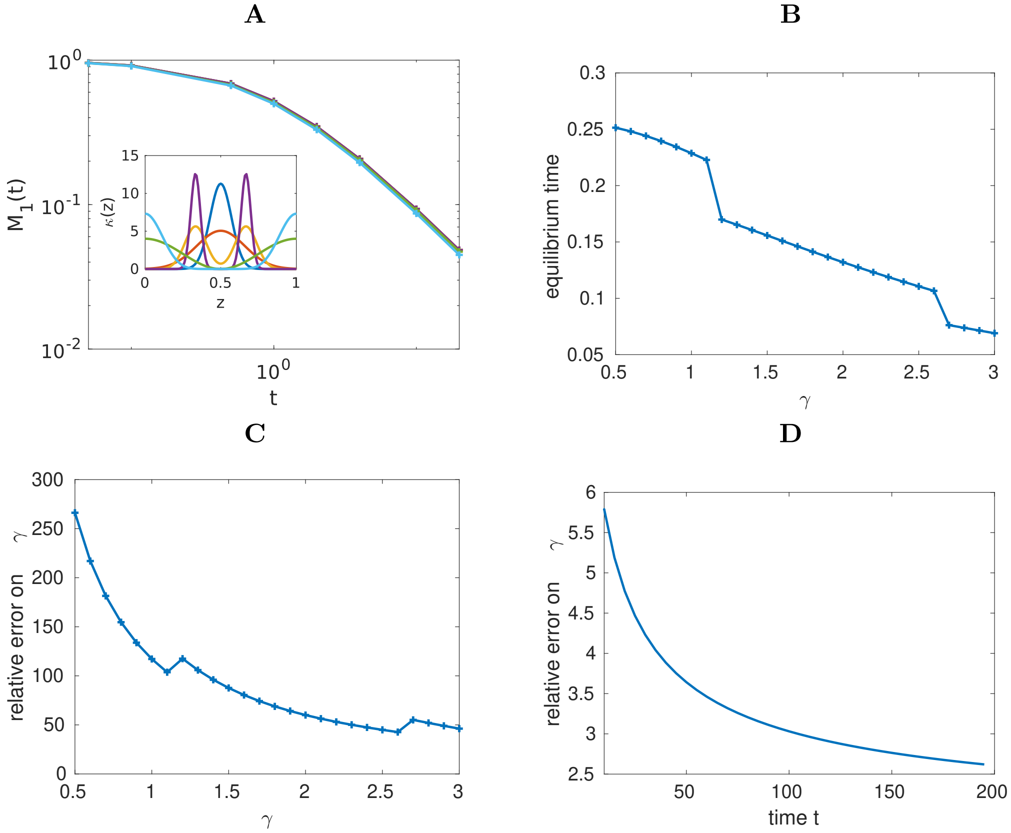


**S 3. Additional figures.** A: Time evolution of $M_1[f(t,.)]$ in a log-log scale for $\gamma = 1$ and for various kernels. The initial condition is a spread gaussian. B: Estimation of the equilibrium time $T_e$ provided by the protocol described in the main text for different values of $\gamma$, the time points being $[0.01, 0.02, 0.03, 0.04, 0.05, 0.1, 0.2, 0.3, 0.4, 0.5, 0.6, 0.7, 0.8, 0.9, 1, 1.5, 2, 4, 5]$. C: The relative error on $\gamma$ as a function of $\gamma$, the time points taken into account being [5,10,...,50]. D: Relative error on $\gamma$ as function of $T_{max}$ the latest time point taken into account, the time points being [5,10,..., $T_{max}$].
