# Supplementary material for: Insights into the dynamic trajectories of protein filament division revealed by numerical investigation into the mathematical model of pure fragmentation
Source: PLoS Comput Biol. 2021 Sep 3;17(9):e1008964. doi: 10.1371/journal.pcbi.1008964 (PMC8462728; doi:10.1371/journal.pcbi.1008964)
Supplement: S4 Fig — (PDF) [file pcbi.1008964.s005.pdf]

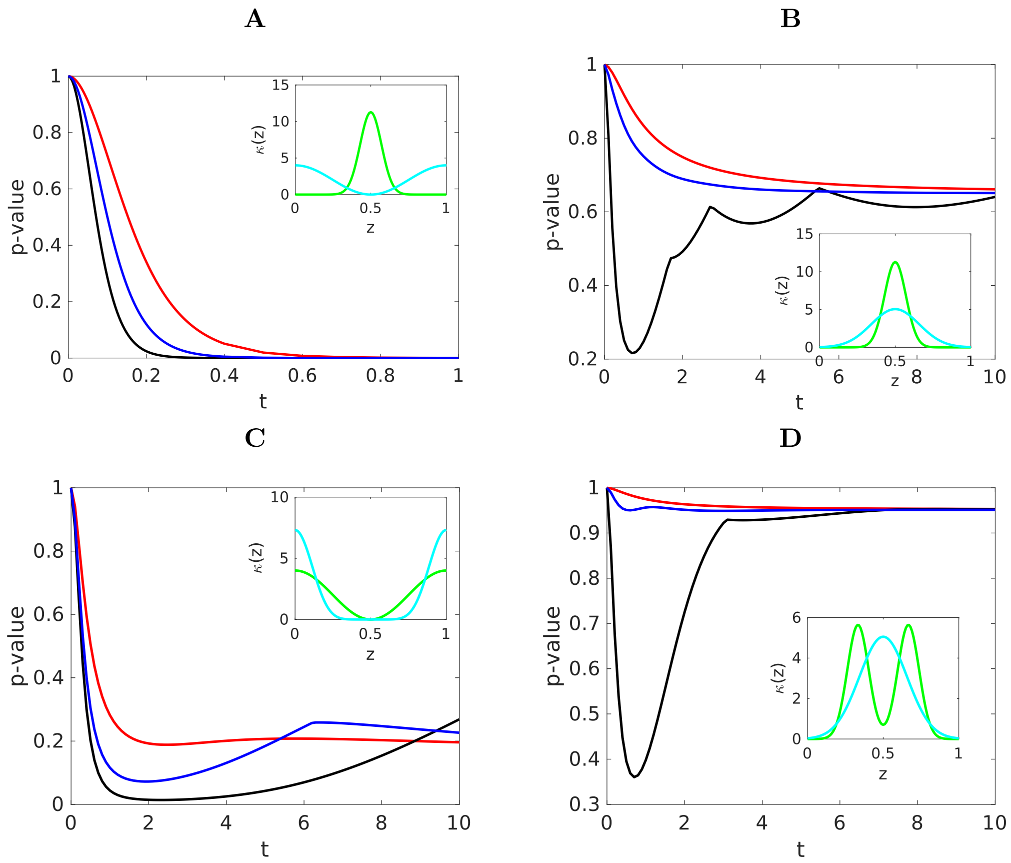

S 4. Plot of the time evolution of the $p-$value corresponding to the null comparison hypothesis $\mathbf{H_0}$, for 3 different initial conditions. Initial conditions: a peaked gaussian (black), a spread gaussian (blue), a decreasing exponential (red). We took $\Delta t = 0.1$. Two different fragmentation kernels cannot be distinguished using late time measurements.
