# Supplementary material for: Insights into the dynamic trajectories of protein filament division revealed by numerical investigation into the mathematical model of pure fragmentation
Source: PLoS Comput Biol. 2021 Sep 3;17(9):e1008964. doi: 10.1371/journal.pcbi.1008964 (PMC8462728; doi:10.1371/journal.pcbi.1008964)
Supplement: S5 Fig — (PDF) [file pcbi.1008964.s006.pdf]

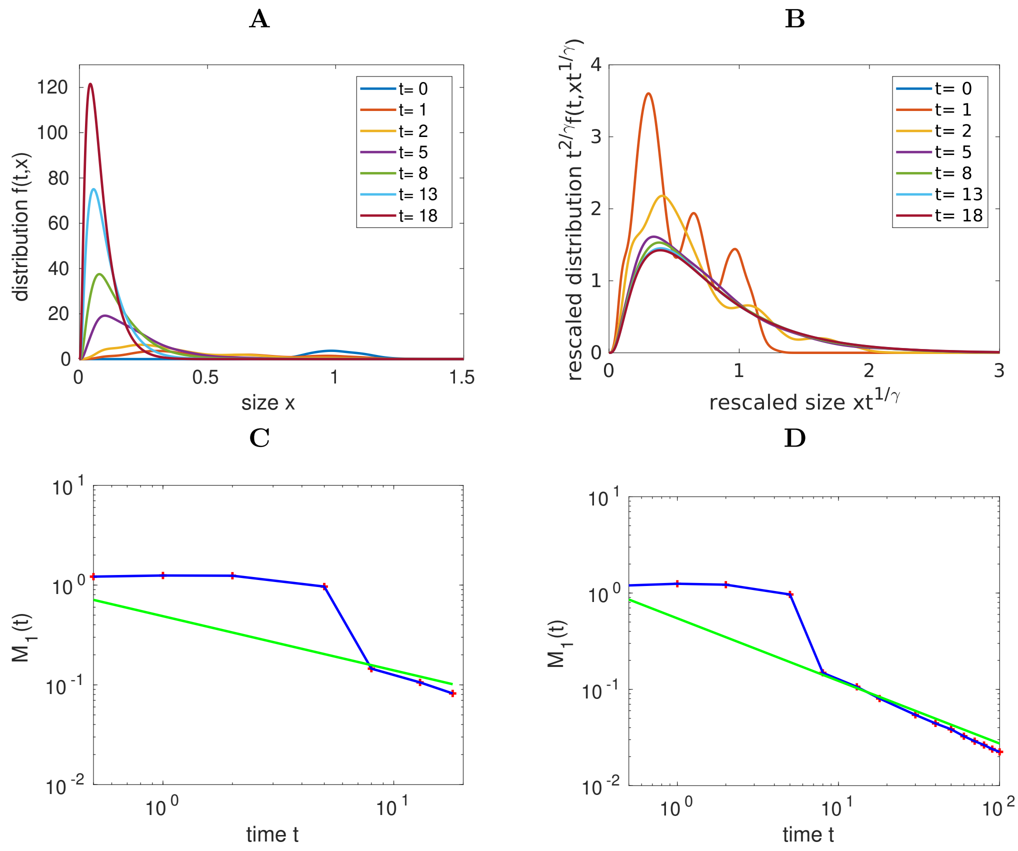

S 5. Illustration of the protocol - Example 2. A: Illustration of on an example of size distribution profiles for $\gamma = 1.3$, $\alpha = 1$, and κ is a two-peaked Gaussian kernel, the initial condition is a spread Gaussian function. Notice that noisy data cannot be represented on a graph, since they formally consists in a sum of delta functions, instead we represent an estimate of the density using the matlab function ksdensity. B: To visualize the profiles more precisely, we rescale the profiles using the real value for γ (i.e $\gamma = 1.3$) and formula (3). What is plotted is for each time t the function $t^{-1/\gamma} f(t, t^{-1\gamma} x)$, where $f(t, .)$ is the distribution profile at time t. This rescaling becomes singular for $t = 0$, hence the absence of rescaling for the initial data. C: Time evolution of the mass M_1 (the data points are red crosses, the solid line blue curve being a linear interpolation), compared to the estimated value γ_e of γ (solid line in green of slope $-1/\gamma_e$.). The code provides us with $\gamma_e = 1.55, \alpha_e = 1.99$ and $T_e = 0.40$. The lack of precision can be interpreted as the lack of information for late time points. D: We add late time data points to visualize the profile of $M_1(t)$.
