# Supplementary material for: Insights into the dynamic trajectories of protein filament division revealed by numerical investigation into the mathematical model of pure fragmentation
Source: PLoS Comput Biol. 2021 Sep 3;17(9):e1008964. doi: 10.1371/journal.pcbi.1008964 (PMC8462728; doi:10.1371/journal.pcbi.1008964)
Supplement: S6 Fig — (PDF) [file pcbi.1008964.s007.pdf]

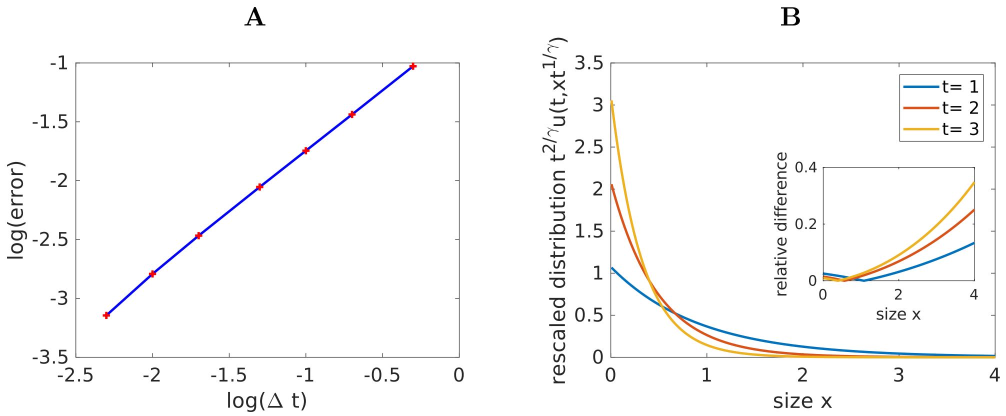


**S 6. Convergence of the numerical scheme.** A: plot of the error at $t = 1$ with respect to a reference solution as a function of $\Delta t$. The plot shows that the scheme is convergent. Parameters: $\gamma = 1.3$, $\alpha = 1$, and $\kappa$ is a two-peaked gaussian kernel. The slope of the straight blue line suggests that the scheme is of order approximately 0.5. B: Comparison between the analytical solution ($\gamma = \alpha = 1$, $s = 0.1$, 4th line of Table 1) and the solution obtained with the numerical scheme. The curves are superimposed and hard to distinguish at the naked eye. The panel inside shows the relative difference between the theoretical and numerical solutions. The relative error becomes large for large $x$ since each solution itself is very small.
