# Supplementary material for: Insights into the dynamic trajectories of protein filament division revealed by numerical investigation into the mathematical model of pure fragmentation
Source: PLoS Comput Biol. 2021 Sep 3;17(9):e1008964. doi: 10.1371/journal.pcbi.1008964 (PMC8462728; doi:10.1371/journal.pcbi.1008964)
Supplement: S7 Fig — (PDF) [file pcbi.1008964.s008.pdf]

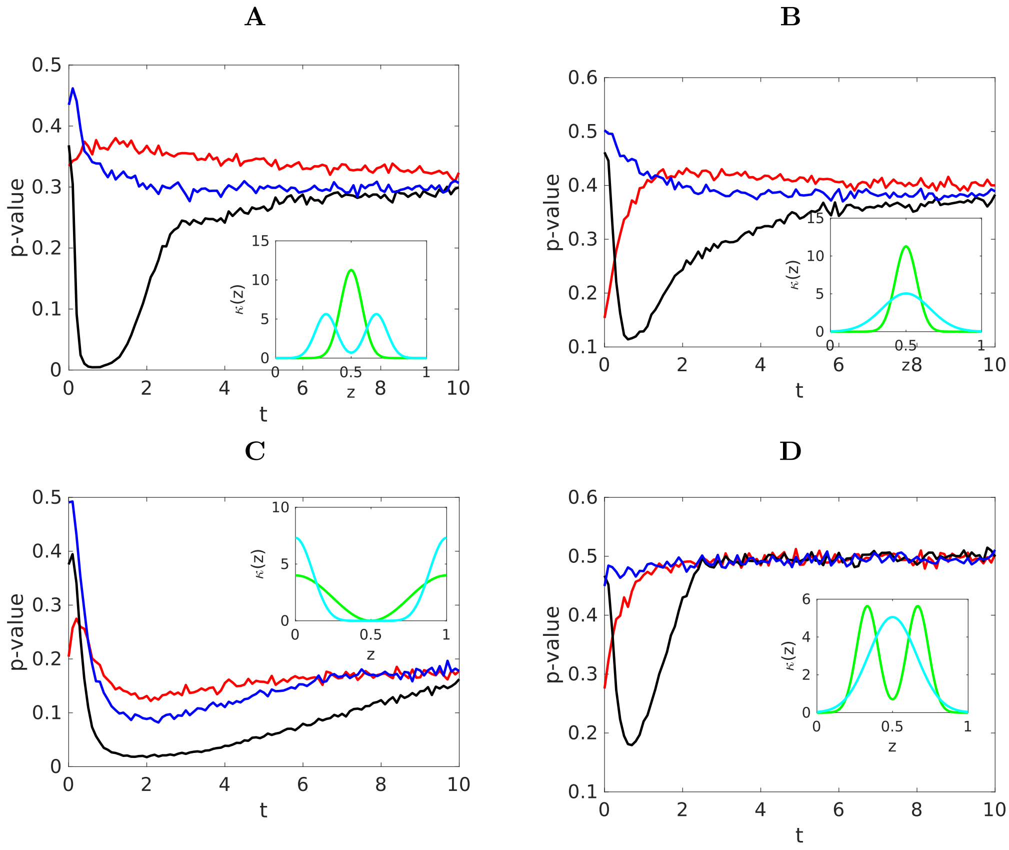

S 7. Plot of the time evolution of the p-value corresponding to the Kolmogorov-Smirnov test for the H_0 hypothesis. Initial condition: a peaked gaussian. Starting from a sample of size $N = 200$ of the initial condition, we estimate the initial distribution, and solve the Eq (3) from that guess. At each time t, a sample of size 200 is gathered for each kernel, and we perform a Kolmogorov-Smirnov test to determine if the underlying distribution are distinguishable. We perform that experiment 500 times and plot the averaged $p-$value. The plots must be put in perspective with those of Fig 4.
