# Supplementary material for: Insights into the dynamic trajectories of protein filament division revealed by numerical investigation into the mathematical model of pure fragmentation
Source: PLoS Comput Biol. 2021 Sep 3;17(9):e1008964. doi: 10.1371/journal.pcbi.1008964 (PMC8462728; doi:10.1371/journal.pcbi.1008964)
Supplement: Attachment — Submitted filename: letter.pdf [file pcbi.1008964.s009.pdf]

PLOS Computational Biology

March 22, 2021

Dear Editor,

We would like to thank the reviewers for taking the time to make a very thorough review. We have taken the advice of all reviewers and have made revisions to the manuscript. Below we outline the changes made by addressing each reviewer's comments. All changes in the manuscript, as well as our point-to-point response, are highlighted in blue.

To reviewer 1:

The paper by Tournus et al provides a very nice study of the dynamics of fragmenting protein filament structures. In particular, the authors have obtained explicit inversion formulas that can be used to extract key information about the rates of protein filament fragmentation in a direct way. The authors also illustrate the power of their method on numerical data and discuss how their results can inform experimental design. Overall, I think that this is a very nice study. I really enjoyed reading this paper, which is well written. The topic is also of great interest due to the relevance of protein filament fragmentation to a range of diseases, particularly in the context of infectious prions. The paper also makes a very nice contribution which is both of theoretical and experimental interest. I therefore recommend this paper for publication, subject to some minor comments outlined here below.

1) The inversion formula for gamma is an asymptotic statement valid for large times. Can the author provide some estimates for the range of times where this formula holds? In other words, how long does one have to measure the length distribution in order to see a straight line of slope q/gamma and extract gamma? Does this estimate depend on the choice of the moment, i.e. q?

The two questions raised by the reviewer are of high importance especially for experimentalists. Determining whether the asymptotic regime has been reached, especially in an experimental setting, is a challenging theoretical question. In this case,, the range of times where our formula holds is expected to depend non-linearly on the parameters α, γ and κ, as well as on the initial condition and on the moment q considered. The rate of convergence towards stationary profile is theoretically polynomial for most initial conditions. However, the information provided is a mere qualitative estimate for the convergence rate. The practical protocol we suggest to experimentalists in this work is as follows: 1. run the simulation (code is made available) using the initial distribution of their sample (can be determined experimentally) and using a first guess for the fragmentation rate and kernel, 2. estimate the time T_e after which the curve $(\log(t), \log(M_1(t)))$ has become a line, 3. perform the experiment until time $5T_e$. To clarify this recommendation, we have added the following sentence in the conclusion of the revised manuscript: "A practical question for experimentalist is to determine whether asymptotic regime has been reached. This a theoretically hard question that can be addressed in a future work. The protocol we suggest to experimentalists is as follows: First: run the simulation (code is made available) using the initial distribution they depart with experimentally and using a first guess for the fragmentation rate adn kernel, second: estimate the time T_e after which the curve $(\log(t), \log(M_1(t)))$ has become a line, and third: perform the experiment until time $5T_e$."

2) The authors have focused on the pure fragmentation kernel. While I am not objecting this choice, I would be very interested in knowing how their results would change in the presence of other aggregation processes, particularly growth? The mathematics in this case would be much more complex because growth introduces new couplings in the aggregation kernel. Would their methods work in this situation as well? Or can the authors suggest some alternative methods/strategies that one may use in this case?

The theoretical results we obtain, and the inversion formula we state are indeed highly dependent of the physical process under study, and on the underlying model.
What is assumed in our paper is that the parameters γ, α and κ are intrinsic characteristics of each type of amyloid fibrils and these parameters are independent of growth. Then the best experiment to identify γ, α and κ is the observation of a population of fibrils of one given type in the absence of growth. The absence

of growth is made possible by guaranteeing experimentally that the monomers in the fibril suspension is depleted and the sample is dilute. To estimate the intrinsic growth rate of a fibril, the protocol we suggest is to first estimate the fragmentation characteristics, and then perform an experiment where fibrils undergo both growth and fragmentation.

The comment by Reviewer 1 may also elude to the unlikely possibility that the fragmentation kernel may be different in presence of growth. If that is the case, we could derive the following. For a generic bounded growth term τ that we can assume depending on the particle sizes x, the model is written as

$$\begin{cases} \dfrac{\partial u}{\partial t}(t,x) + \dfrac{\partial}{\partial x}\left(\tau(x)u(t,x)\right) = -\alpha x^\gamma u(t,x) + \alpha \displaystyle\int_x^\infty \frac{1}{y}\kappa\left(\frac{x}{y}\right) y^\gamma u(t,y)dy, \quad t>0,\ x\geq 0, \\ u(0,x) = u^0(x), \quad x>0. \end{cases} \tag{1}$$

The asymptotic behaviour of the equation above is well-understood [1]: under reasonable assumptions on the parameters, the solution u converges after rescaling toward a stationary profile N in the sense

$$e^{-\lambda t}u(t,x) \underset{t\to\infty}{\rightarrow} N(x), \tag{2}$$

and where N is the solution of

$$\begin{aligned} &\frac{\partial}{\partial x}\left(\tau(x)N(x)\right) + \lambda N(x) = -\alpha x^\gamma N(x) + \alpha \int_x^\infty \frac{1}{y}\kappa\left(\frac{x}{y}\right) y^\gamma N(y)dy, \quad x \geq 0, \\ &\tau(0)N(0) = 0, \quad \int_0^\infty N(y)dy = 1, \qquad N\geq 0. \end{aligned} \tag{3}$$

The strategy to recover the parameters depends on the context and on the type of data we can obtain. Let us consider that we can access the measurement of the size distribution at several time points. If we assume that the growth rate τ is known, then, here is what could be a strategy to obtain the other parameters:

1. Estimate λ. Contrarily to the pure fragmentation equation where convergence is polynomial with a rate depending explicitly on the parameter γ, here, the convergence is exponential with a parameter λ that is defined as the first eigenvalue of a linear operator, and then, that is not directly accessible. Even if λ is not a parameter of equation (1) per se, its value is needed to obtain the values of α, γ and κ. If we only access the size distribution, i.e. the distribution f defined as

$$f(t,x) = \frac{u(t,x)}{\int_{\mathbb{R}^+} u(t,x)dx},$$

where u is the solution to (1), then the asymptotic behaviour (2) is not helping. Instead, a possible strategy to estimate λ would be to follow a result in [2]. Multiplying equation(3) by x and integrating over $\mathbb{R}^+$ provides us with the formula

$$\lambda = \frac{\int_{\mathbb{R}^+} \tau(x)N(x)dx}{\int_{\mathbb{R}^+} xN(x)dx}. \tag{4}$$

2. Equation (3) in Mellin coordinates. We denote by $M[N]$ and $M[\tau]$ the respective Mellin transforms of N and τ. Equation (3) in Mellin coordinates becomes

$$(s-1)M[\tau N](s-1) + \lambda M[N](s) = \alpha\gamma(K(s)-1)M[N](s+\gamma) \qquad \forall s\in\mathbb{C}, \quad 1<\Re e(s)\leq 2. \tag{5}$$

3. Estimate γ. To estimate γ, one strategy could be to use a characterization similar to the characterization proved in [3]. This characterization relies on the knowledge of high order moments of the solution, which are difficult to measure in practice.

4. Estimate for α. Using $\int \kappa(z)dz = 2$ (binary fission), i.e. $M[\kappa](1) = 2$, we obtain, similarly to what we had in our paper

$$\alpha = \frac{M[N](1)}{\gamma M[N](1+\gamma)}$$

5. Estimate κ. Once α and γ are known, we may formally divide Equation (5) by $M[N](s+\gamma)$ and obtain

$$K(s) = 1 + \frac{(s-1)M[\tau N](s-1) + \lambda M[N](s)}{\alpha\gamma M[N](s+\gamma)}.$$

If the growth rate τ is also unknown, it is not clear if and how the 4 parameters α, γ, τ and κ can be extracted from the measurement of the size distribution at several time points.
Therefore, we emphasize that all the qualitative conclusions we obtained numerically cannot be directly extended to the growth-fragmentation equation if the parameters governing fragmentation and growth are interdependent. To clarify on this point, we have added the following sentence in the conclusion of the revised manuscript:. "What is assumed in the present paper is that the parameters γ, α and κ are intrinsic characteristic of each type of amyloid fibril. Then an adequate experiment to identify γ, α and κ is the observation of a population of fibrils of one given type in the absence of growth. Absence of growth is made possible by guaranteeing experimentally that the suspension is dilute enough. It may be of interest to also estimate the intrinsic growth rate of the fibrils under study. The protocol we suggest is to separate the experiments and to first estimate the fragmentation characteristics as presented in this paper, and then focus on the growth rate. ".

Some typos: 1) Page 10, line 8: Should $E(\alpha)$ be related to $|\alpha - \alpha_e|$ rather than $|\alpha - \gamma_e|$?

Indeed, this is a typo, that has been corrected.

To reviewer 2:

The paper is concerned with pure fragmentation processes as it occurs for polymeric protein filaments and more precisely on the inverse problem. The question is to recover the division rate and division kernel from the observation of the dynamics of filament size distribution. Being given the experimental set up, the continuous fragmentation model is used. To recover the division rate, the theory is built on the, by now, well-known self-similar profile achieved in long times. The method to build the division kernel is based on the Mellin transform according to a theory developed earlier, in particular by the authors. The shape of kappa, influences in a direct and remarkable way the shape of the asymptotic self-similar profile.

Departing findings are that neither the stationary state nor the knowledge of the mere moments are sufficient to recover the kernel kappa. An inversion procedure has to be devised which is performed here and allows to recover the fibrils distribution dynamics. A remarkable conclusion is that the kernels can be two different types (A or B here). The paper is careful about real experimental data and taking into account uncertainties in measurements and the challenge of diminishing filament size along time.

After the theoretical background, the paper describes inverse problem procedure, the needed experimental data and their analysis. It uses a numerical code validated in comparison with another code and vs analytical solutions. A statistical test is used to possibly distinguish the expected kernels.

The problem of understanding the size distribution is important for several areas as cell size distribution and fibrils dynamics for neuro-degenerative diseases. Based on recent discoveries and methods from mathematical analysis of the relevant models, the present contribution is an important step in the concrete determination of the model parameters.

Minor remarks

p6: scission —¿ Fission ?

We modified it.

p7: there is no need of this long discussion to discover that alpha is a time scale. This is obvious from (3).

As mathematicians, we agree with the reviewer, but since the journal targets a large audience, we cannot assume that every reader is familiar with partial differential equations and their interpretations.

Sometimes it is written "on Figure" or "in Figure". The former being more correct.

We replace all the occurrences of 'In Figure' by 'On Figure'.

After (13) change coma to dot.

We modified it.

(17) check the inequalities (how can mass change after the steady state is reached) and the space next line

The total mass of the system can vary even after the steady regime is reached, because steady regime

does not mean convergence of the distribution f towards a steady state, but convergence of the rescaled distribution $t^{-1/\gamma} f(t, t^{-1\gamma}.)$ towards a steady profile g. It was not clear enough in the first version of our paper, so we replaced "when the steady profile considered reached" by "when the asymptotic line is considered reached i.e. the rescaled distribution $x \to t^{-1/\gamma} f(t, t^{-1\gamma} x)$ has aligned with the steady profile g".

p12: n cannot be both the number of measurements and the generic times

Indeed,we re-indexed the generic time with the letter j.

p14: these explicit solutionS

We modified it.

Bibliography needs some uniformisation; in [33] critIcal

We uniformized the bibliography and modified ref. 33.

To reviewer 3:

This paper analyzed the ability of a previously published fragmentation model to fit real experimental data by developing several new algorithms, including a numerical method and an application of statistical hypothesis testing. The authors provide a "type" of sensitivity analysis (see my first comment below) and describe a new numerical algorithm to estimate the fragmentation rate and kernel from experimental measurements. They also derive inversion formulas to recover the three key parameters from experimental measurements. The results of this study are novel and important because the focus is on performing a practical inverse problem with respect to what can actually be experimentally measured, namely the particle length distribution at various time points in a fragmentation experiment. Another important result is that their method doesn't rely on traditional parameter estimation methods, but instead an analytic inversion formula is derived that expresses the three key model parameters as functions that can be directly computed from the solution to the fragmentation equation. Given the practical and experiment focused nature of these results, it is likely that this work will enable the design of future experiments whose purpose is to investigate the stability of protein filaments and the ensuing fragmentation process. Before publication, I suggest two items be addressed:

1) The "sensitivity analysis" presented is not rigorous (or what would typically be expected when an applied math paper refers to "sensitivity analysis") and instead the results presented are, as stated in the paper, a numerical investigation of the influence of the three key parameters. For example, a rigorous "local" sensitivity analysis would include the computation of the partial derivative of the output, i.e., the rescaled distribution, with respect to the parameter of interest, evaluated at some chosen parameter value. A "global" sensitivity analysis would include something like Morris screening or latin hypercube sampling to compute sensitivity over an entire parameter domain. Instead, the authors are changing the value of gamma and alpha and plotting the rescaled distribution at these values. My suggestion is to rename the analysis to reflect that the authors are exploring how the solutions change as they vary parameters over a chosen domain, since the name "sensitivity analysis" is widely used in the applied math literature to be either local or global types mentioned above, and this may be confusing to the reader. Alternatively, the authors could perform either a local or global sensitivity analysis and include the results.

We renamed what we called in the previous version a 'sensitivity' analysis, and we refer to it as 'a numerical investigation of the influence of the three key parameters'.

2) While the introduction is very well written, and the results and methods include very thorough details, the conclusions section could be expanded to include a discussion or examples for the two points mentioned at the end of the introduction. Namely, (1) that "the method and the analysis presented here are general and can be useful in other contexts" and (2) "the mathematical results will inform the design of experiments tailored to evaluate and compare the dynamical stabilities of protein filaments".

We have added new text in the conclusion section of our revised manuscript as suggested by the reviewer. (1) Fragmentation equation is used in many different applications (not only fibrils filaments), for which our inverse method can apply to. In particular, with a modern experimental approach that would provide size distribution profiles, the fragmentation rate and kernel can be obtained for polymers. (2) This suggests that the experiments that provide the best data to estimate γ and α are long time experiments starting with any initial distribution: several data points are needed after asymptotic regime is reached. On the contrary, to

estimate the fragmentation kernel κ, the experiment should rather start with a highly peaked distribution, which means that all fibrils should have approximately the same length, and the time measurements of interest are short time points (before asymptotic regime is reached). Such an initial distribution is very complicated to obtain experimentally, and we explore in a future work how the spread of the initial distribution affects the estimate of κ.

[1]. B. Perthame. *Transport equations in biology.* Frontiers in Mathematics. Birkhäuser Verlag, Basel, 2007. ISBN 978-3-7643-7841-7; 3-7643-7841-7.

[2]. P. Michel, S. Mischler, and B. Perthame. General relative entropy inequality: an illustration on growth models. *Journal de Mathématiques Pures et Appliquées*, 84(9):1235 – 1260, 2005. ISSN 0021-7824. http://dx.doi.org/10.1016/j.matpur.2005.04.001.

[3]. M. Doumic, M. Escobedo, and M. Tournus. Estimating the division rate and kernel in the fragmentation equation. *Ann. Inst. H. Poincaré Anal. Non Linéaire*, 35(7):1847–1884, 2018. ISSN 0294-1449. 10.1016/j.anihpc.2018.03.004. URL https://doi.org/10.1016/j.anihpc.2018.03.004.
